# Bioinformatic analysis reveals potential relationship between chondrocyte senescence and protein glycosylation in osteoarthritis pathogenesis

**DOI:** 10.3389/fendo.2023.1153689

**Published:** 2023-05-17

**Authors:** Makoto Yoshimoto, Koki Sadamori, Kazuya Tokumura, Yuki Tanaka, Kazuya Fukasawa, Eiichi Hinoi

**Affiliations:** ^1^ Department of Bioactive Molecules, Pharmacology, Gifu Pharmaceutical University, Gifu, Japan; ^2^ United Graduate School of Drug Discovery and Medical Information Sciences, Gifu University, Gifu, Japan; ^3^ Center for One Medicine Innovative Translational Research, Division of Innovative Modality Development, Gifu University, Gifu, Japan

**Keywords:** osteoarthritis, chondrocyte, cellular senescence, protein glycosylation, gene expression omnibus

## Abstract

Osteoarthritis (OA) is the most common degenerative and progressive joint disease. Cellular senescence is an irreversible cell cycle arrest progressive with age, while protein glycosylation is the most abundant post-translational modification, regulating various cellular and biological pathways. The implication of either chondrocyte senescence or protein glycosylation in the OA pathogenesis has been extensively and individually studied. In this study, we aimed to investigate the possible relationship between chondrocyte senescence and protein glycosylation on the pathogenesis of OA using single-cell RNA sequencing datasets of clinical OA specimens deposited in the Gene Expression Omnibus database with a different cohort. We demonstrated that both cellular senescence signal and protein glycosylation pathways in chondrocytes are validly associated with OA pathogenesis. In addition, the cellular senescence signal is well-connected to the O-linked glycosylation pathway in OA chondrocyte and vice-versa. The expression levels of the polypeptide N-acetylgalactosaminyltransferase (GALNT) family, which is essential for the biosynthesis of O-Glycans at the early stage, are highly upregulated in OA chondrocytes. Moreover, the expression levels of the GALNT family are prominently associated with chondrocyte senescence as well as pathological features of OA. Collectively, these findings uncover a crucial relationship between chondrocyte senescence and O-linked glycosylation on the OA pathophysiology, thereby revealing a potential target for OA.

## 1 Introduction

Osteoarthritis **(**OA) is the most common degenerative and progressive joint disease, frequently leading to functional disability and chronic pain in the elderly (1, 2). The economic toll and prevalence of OA are steadily rising worldwide, making it a representative public health concern for the coming decade (3, 4).. OA is characterized by degeneration of articular cartilage, synovial inflammation, osteophyte formation, and subchondral sclerosis (5, 6). Although much attention has been focused on the mechanisms of initiation and progression of OA, the precise pathogenesis of OA remains unclear, leading to no availability of disease-modifying osteoarthritis drugs (DMOADs) (7, 8). The initiation and progression of OA are induced and regulated by numerous factors, including joint injury, obesity, inflammation, gender, heredity, aging, and cellular senescence (9, 10).

Cellular senescence, an irreversible cell cycle arrest that various intrinsic and extrinsic factors can induce in normal cells, is recently shown to contribute to the OA phenotype (11, 12). Senescent chondrocytes were shown to accumulate in OA cartilage lesions and release the senescence-associated secretory phenotype (SASP) factors into the surrounding microenvironment (13, 14). The OA development was ameliorated by the genetic and pharmacological removal of senescent chondrocytes in the OA mouse model, where intraarticular transplantation of senescent chondrocytes-induced cartilage degeneration suggested that senescent chondrocytes could be an effective target of OA prevention and treatment (15, 16). Protein glycosylation plays a pivotal role in cartilage homoeostasis as well as degeneration. Notably, clinical investigations of OA patients and basic research utilizing animal models have demonstrated a relationship between abnormal glycosylation and the onset and progression of OA.

Protein glycosylation, well-known as one of the major post-translational modifications, regulates various cellular and biological pathways, including signal transduction, proliferation, differentiation, and survival (17, 18). It is classified into N*-*linked glycosylation, O*-*linked glycosylation, C-mannosylation, phospho-glycosylation, and glypiation (19, 20). Aberrant glycosylation is associated with multiple human diseases, including congenital disorders of glycosylation (CDGs), cancer, and autoimmune disease (17, 21). Protein glycosylation is vital in physiological cartilage homeostasis and pathological cartilage degeneration (22–25). Alternations in high-mannose type N-glycans were observed in both human OA cartilage and degraded mouse cartilage, along with the increase of β1,2N-acetylglucosaminyltransferase I (GlcNAc-TI) (23). Moreover, O-linked N-acetylglucosamine (O-GlcNAc) protein modification is increased in the cartilage of OA patients (24).

Although extensive studies have been conducted to reveal the implication of either chondrocyte senescence or protein glycosylation in the initiation and progression of OA, no evidence is available regarding the pivotal relationship between cellular senescence signals and protein glycosylation pathway in chondrocytes on the pathogenesis of OA.

Thus, in this study, we aimed to investigate the possible relationship in OA chondrocytes using single-cell RNA sequencing (scRNA-seq) datasets of clinical OA specimens deposited in the Gene Expression Omnibus (GEO) database.

## 2 Materials and methods

### 2.1 Information on scRNA-seq data

sc-RNA-seq data, GSE104782 and GSE169454 (26, 27), were obtained from the Gene Expression Omnibus of the National Center for Biotechnology Information (https://www.ncbi.nlm.nih.gov/geo/). The GSE104782 dataset contains 1600 chondrocytes of 10 patients with OA undergoing knee arthroplasty surgery, and cells are labeled with OA grades according to the OARSI grading system (grade 0; *n* = 320, grade 1; *n* = 320, grade 2; *n* = 320, grade 3; *n* = 320, grade 4; *n* = 320). These scRNA-seq libraries were generated using a Kapa Hyper Prep Kit (Kapa Biosystems, Wilmington, MA, USA) and sequenced using the Illumina HiSeq 4,000 platform (Novogene, Beijing, China) with a read length of 150 bp. The GSE169454 dataset contains 7 scRNA-seq dates. These scRNA-Seq libraries were generated using the 10X Genomics Chromium Controller Instrument and Chromium Single Cell 3’ V3 Reagent Kits (10X Genomics, Pleasanton, CA, USA) and sequenced using the Illumina HiSeq 4,000 platform (Novogene) with a read length of 150 bp.

### 2.2 Processing of scRNA-seq data

The data were analyzed using the “Seurat” package (ver 4.2.0) of the R software (ver 4.0.2). First, sc-RNA-seq data, GSE104782 and GSE169454 (26, 27), were read with the Read10X function. In the preprocessing of the GSE104782 dataset, cells with > 7500 and < 200 expressed genes were considered low-quality cells and were removed with the subset function. Accordingly, 1457 chondrocytes (grade 0; *n* = 262, grade 1; *n* = 302, grade 2; *n* = 305, grade 3; *n* = 284 or grade 4; *n* = 304) were used for further analysis. In the preprocessing of the GSE169454 dataset, cells with > 6000 and < 500 expressed genes with a proportion of mitochondrial genes > 25% were considered low-quality cells and were removed with the subset function. For each dataset, normalization was performed with the SCTransform function with the removal of the mitochondrial mapping percentage and the method set of glmGamPoi. To remove the batch effects, integration of the 7 sample datasets in GSE169454 was performed using reciprocal PCA (RPCA) including the process of SelectIntegrationFeatures, PrepSCTIntegration, RunPCA, FindIntegrationAnchors, and IntegrateData function. Accordingly, 66795 chondrocytes (cartilages of 3 patients without OA, *n* = 8887; and cartilages of 4 patients with OA, *n* = 57908) were used for subsequent bioinformatic analysis.

### 2.3 Identification of differentially expressed genes (DEGs) and gene set enrichment analysis (GSEA)

DEGs were identified among 220 human glycosylation-related genes registered in GGDB (Glycogene Database) using Wilcoxon’s rank-sum test by the “presto” package (ver 1.0.0) with the wilcoxauc function (*P* < 0.05). GSEA was performed using the “clusterProfiler” package (ver 3.18.1). GSEA results are evaluated based on an enrichment score, which represents the extent to which a given gene set is overrepresented at the top or bottom of a ranked gene list. The normalized enrichment score (NES) considers differences in gene set size and correlation between gene and expression data sets. Other factors that are also reflected in the analysis include the *P*-value, which indicates the significance of the enrichment score, and the false discovery rate (FDR), which indicates the probability of a false positive result (28, 29). GSEA was performed to compare gene set enrichment between the two groups. The area under the receiver operator curve (AUC) was calculated using Wilcoxon’s rank-sum test with the wilcoxauc function. Gene lists in descending order of AUC were created, and GSEA was performed using the gene lists with the GSEA function (parameters: minGSsize = 5, maxGSsize = 500, eps = 0, pvalueCutoff = 1.00). The gene sets were obtained from the MSigDB databases (http://www.gsea-msigdb.org/gsea/msigdb/index.jsp) using the msigdbr (ver 7.5.1) package with the msigdbr function. The HALLMARK gene sets were obtained from the H collection of the MSigDB databases, the GOBP gene sets from the C5 collection, and the REACTOME gene sets from the C2 collection. Gene sets with NES > 0 and *P* < 0.05 were considered enriched. The visualization of GSEA results was performed using the “enrichplot” package (ver 1.10.2) with the gseaplot2 function. To investigate the association of GALNT family gene expression with pathological features of OA and chondrocyte senescence, GSEA was performed on two groups of chondrocytes (the top 25% and bottom 25% of the expression level of the GALNTs-family in OA chondrocytes at all grades are defined as GALNTs-expression^high^ chondrocytes (n = 364) and GALNTs-expression^low^ chondrocytes (n = 364), respectively).

Single sample GSEA (ssGSEA) was performed using the “GSVA” package (ver 1.38.2) with the gsva function. ssGSEA is an extension of GSEA that allows separate enrichment scores to be calculated for each sample and gene set pair. Each ssGSEA enrichment score represents the extent to which genes within a particular gene set are cumulatively upregulated or downregulated in a given sample (30, 31). ssGSEA on the scRNA-seq dataset (GSE104782) was performed to compare gene set enrichment for each OA stage and divide the chondrocytes into two groups in subsequent analysis (senescence^high^ OA chondrocytes; *n* = 729, senescence^low^ OA chondrocytes; *n* = 364, O-linked glycosylation^high^ OA chondrocytes; *n* = 729, O-linked glycosylation^low^ OA chondrocytes; *n* = 364, N-linked glycosylation^high^ OA chondrocytes; *n* = 729, N-linked glycosylation^low^ OA chondrocytes; *n* = 364). For ssGSEA on the scRNA-seq dataset (GSE169454), OA chondrocytes were divided into two groups (senescence^high^, senescence^low^, O-linked glycosylation^high^, O-linked glycosylation^low^, N-linked glycosylation^high^, N-linked glycosylation^low^ OA chondrocytes; *n* = 5790, respectively). The visualization of ssGSEA results was performed using the “ggplot2” package (ver 3.3.6) with the ggplot function.

### 2.4 Statistical analysis

Statistical analysis between the two groups was performed using Wilcoxon’s rank-sum test for DEG and permutation test for GSEA. Statistical analysis between the three groups was performed using Wilcoxon’s rank-sum test followed by Bonferroni’s correction. Statistical significance was set as *P* < 0.05. Significance levels are indicated by asterisks (*P < 0.05, **P < 0.01, ***P < 0.001).

## 3 Results

### 3.1 Cellular senescence signal and protein glycosylation pathways in chondrocytes are associated with the OA pathogenesis

**Figure 1:**
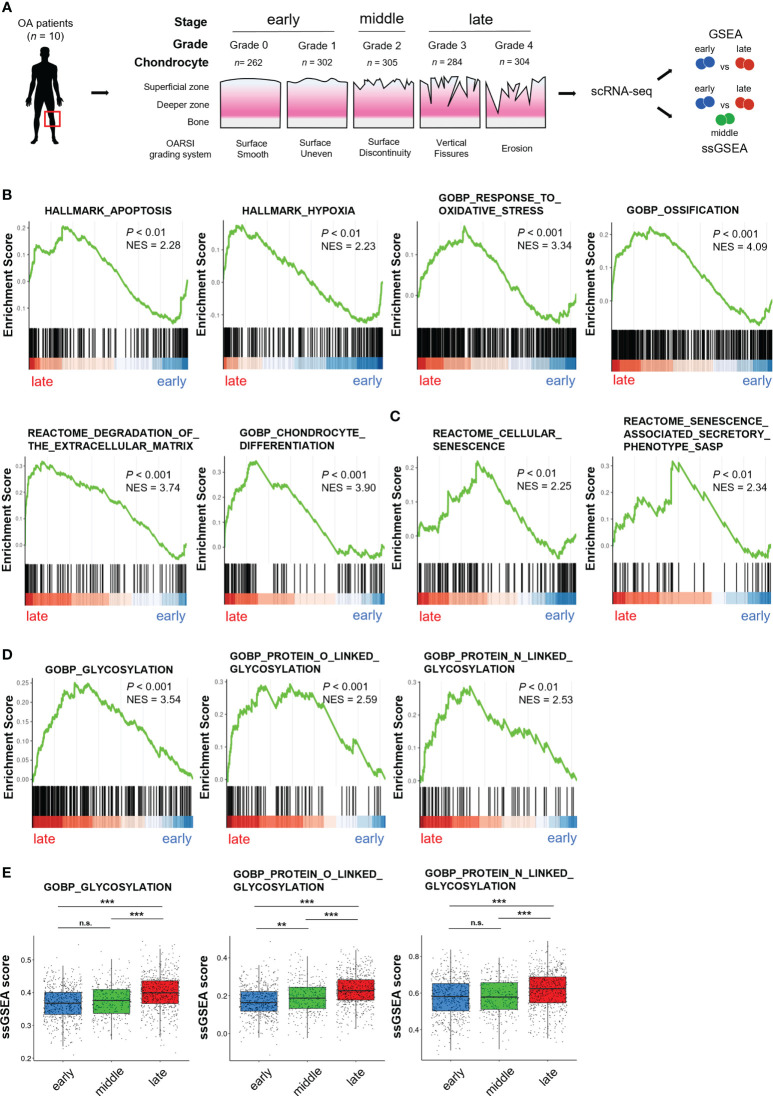
Cellular senescence signal and protein glycosylation pathways are enriched in OA chondrocytes at the late stage. **(A)** Schematic diagram of sample information and single-cell RNA-seq analysis of the GSE104782 dataset. Chondrocytes were harvested from ten patients with OA (early [grade 0; *n* = 262, and grade 1; *n* = 302], middle [grade 2; *n* = 305], and late [grade 3; *n* = 284, and grade 4; *n* = 304] stages). **(B–D)** Gene set enrichment analysis (GSEA) of OA chondrocytes. **(B)** “apoptosis”, “hypoxia”, “oxidative stress”, “ossification,” “ECM degradation” and “differentiation”, gene sets. **(C)** “cellular senescence” and “SASP” gene sets. **(D)** “glycosylation”, “O-linked glycosylation,” and “N-linked glycosylation” gene sets. **(E)** Single sample GSEA (ssGSEA) scores of glycosylation pathways in each stage of OA (**P < 0.01, ***P < 0.001, n.s., not significant).

We first used a scRNA-seq dataset derived from clinical OA specimens deposited on the GEO database (GSE104782) to profile the properties of OA chondrocytes at different stages (26). We analyzed chondrocytes obtained from the articular cartilage at the OA early stages (grades 0 and 1) and the late stages (grades 3 and 4) based on the gene set associated with OA pathogenesis by GSEA (**Figure 1A**). We determined the enrichment of gene sets involved in “apoptosis,” “hypoxia,” “oxidative stress,” “ossification,” “ECM degradation,” and “differentiation,” which have a robust association with OA pathogenesis (1, 5), in chondrocytes of the late stage OA, confirming the validity of the scRNA-seq dataset used for our further analyses (**Figure 1B**).

Chondrocyte senescence has emerged as a fundamental mechanism which substantially contributes to OA phenotype (11, 12). GSEA revealed that gene sets involved in “cellular senescence” and “SASP” were enriched in chondrocytes at the late-stage OA (**Figure 1C**). Aberrant glycosylation is linked to OA development in the clinical specimen and animal models (23, 24). Among the gene sets associated with glycosylation, GSEA revealed significant enrichment for gene sets related to the “glycosylation,” “O-linked glycosylation,” and “N-linked glycosylation” in chondrocytes at the late stage OA (**Figure 1D**). Moreover, the enrichment of gene set related to “O-linked glycosylation” was positively associated with increased OA grades (early (grade 0 and 1), middle (grade 2) and late (grade 3 and 4) stages) by ssGSEA, while gene sets related to “glycosylation” and “N-linked glycosylation” were not significantly altered between the early stage and the middle stage (**Figure 1E**).

These results indicate that chondrocyte senescence signal and protein glycosylation pathways are linked to the OA phenotypes.

### 3.2 Cellular senescence signal is associated with O-linked glycosylation pathway in OA chondrocyte, and vice versa

Both cellular senescence and protein glycosylation in chondrocytes are linked to the OA phenotype, as described in **Figure 1**. However, the possible relationship between chondrocyte senescence and protein glycosylation in the pathophysiology of OA remains undefined. We, therefore, determined whether the cellular senescence signal was associated with the protein glycosylation pathways in OA chondrocytes by performing GSEA on the scRNA-seq dataset (GSE104782).

**Figure 2:**
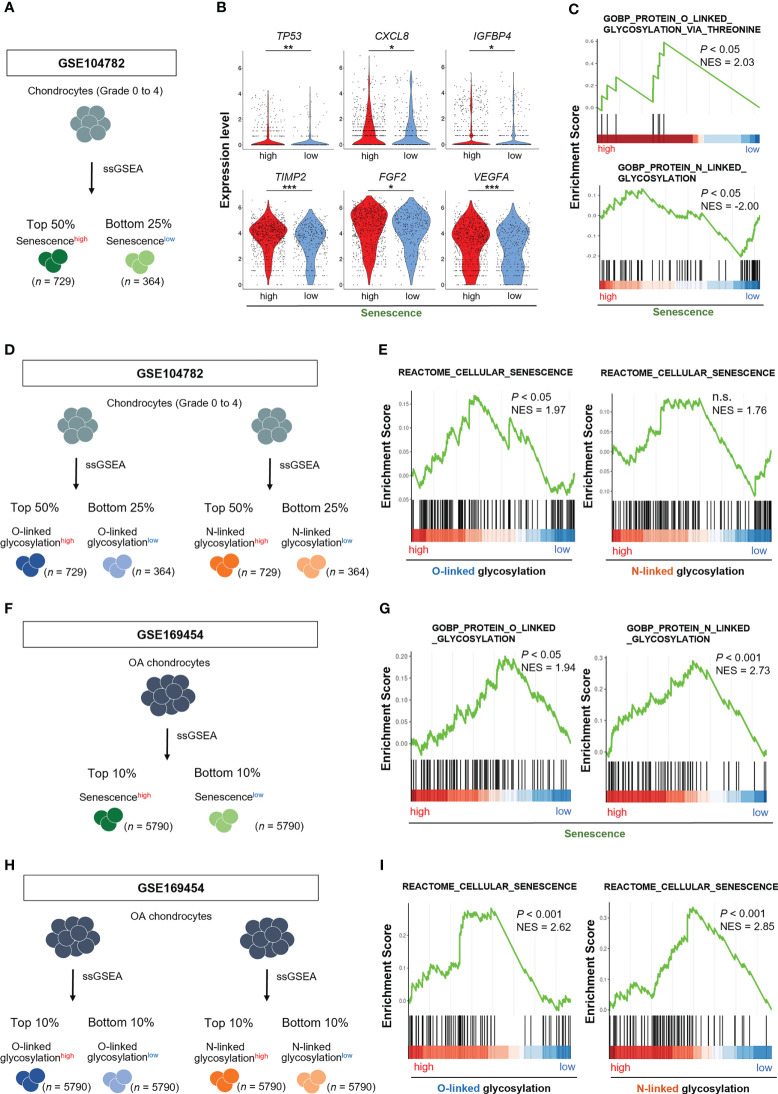
O-linked glycosylation pathway is enhanced in senescence^high^ OA chondrocytes, and vice versa. **(A)** Schematic diagram of chondrocyte classification by ssGSEA of genes associated with “cellular senescence” in GSE104782 (senescence^high^ OA chondrocytes [*n* = 729], senescence^low^ OA chondrocytes [*n* = 364]). **(B)** Expression levels of cellular senescence and SASP marker genes in senescence^high^ and senescence^low^ OA chondrocytes. **(C)** GSEA of “O-linked glycosylation” and “N-linked glycosylation” gene sets in senescence^high^ OA chondrocytes. **(D)** Schematic diagram of chondrocyte classification by ssGSEA of genes associated with “O-linked glycosylation” and “N-linked glycosylation” in the GSE104782 dataset (glycosylation^high^ OA chondrocytes [*n* = 729] and glycosylation^low^ OA chondrocytes [*n* = 364]). **(E)** GSEA of “cellular senescence” gene set in glycosylation^high^ OA chondrocytes. **(F)** Schematic diagram of chondrocyte classification by ssGSEA of genes associated with “cellular senescence” in GSE169454 (senescence^high^ OA chondrocytes [*n* = 5790], senescence^low^ OA chondrocytes [*n* = 5790]). **(G)** GSEA of “O-linked glycosylation” and “N-linked glycosylation” gene sets in senescence^high^ OA chondrocytes. **(H)** Schematic diagram of chondrocyte classification by ssGSEA of genes associated with “O-linked glycosylation” and “N-linked glycosylation” in the GSE169454 dataset (glycosylation^high^ OA chondrocytes [*n* = 5790] and glycosylation^low^ OA chondrocytes [*n* = 5790]). **(I)** GSEA of “cellular senescence” gene set in glycosylation^high^ OA chondrocytes. *P < 0.05, **P < 0.01, ***P < 0.001.

We divided chondrocytes obtained from all stages (grade 0 to 4) into two groups, Senescence-signal^high^ and Senescence-signal^low^ chondrocytes, based on the gene set associated with “cellular senescence (REACTOME_CELLULAR_SENESCENCE)” by ssGSEA (**Figure 2A**). The expression levels of *TP53*, *CXCL8*, *IGFBP4*, *TIMP2*, *FGF2*, *VEGFA*, which are known as cellular senescence and SASP markers, were significantly higher in Senescence-signal^high^ chondrocytes, validating our classification method by ssGSEA (**Figure 2B**). GSEA revealed that the gene set related to the O-linked glycosylation *via* threonine was significantly enriched in Senescence-signal^high^ chondrocytes, whereas the N-linked glycosylation-related gene set was significantly enriched in Senescence-signal^low^ chondrocytes (**Figure 2C**).We then divided into O-linked or N-linked glycosylation-pathway^high^ and O-linked or N-linked glycosylation-pathway^low^ chondrocytes, based on the gene sets associated with O-linked or N-linked glycosylation (GOBP_PROTEIN_O_LINKED_GLYCOSYLATION or GOBP_PROTEIN_N_LINKED_GLYCOSYLATION) by ssGSEA (**Figure 2D**), and noted the enrichment for the gene set related to the “cellular senescence” in O-linked glycosylation-pathway^high^ chondrocytes but not in N-linked glycosylation-pathway^high^ chondrocytes (**Figure 2E**).

To confirm the results obtained from GSE104782, we analyzed the different scRNA-seq dataset (GSE169454). We divided OA chondrocytes into two groups, Senescence-signal^high^ and Senescence-signal^low^ chondrocytes, based on the gene set associated with “cellular senescence” by ssGSEA (**Figure 2F**). GSEA revealed the enrichment for the gene sets related to the O-linked and N-linked glycosylation in Senescence-signal^high^ chondrocytes (**Figure 2G**). We further divided into O-linked or N-linked glycosylation-pathway^high^ and O-linked or N-linked glycosylation-pathway^low^ chondrocytes, based on the gene sets associated with O-linked or N-linked glycosylation by ssGSEA (**Figure 2H**). GSEA revealed the enrichment for the gene set related to the “cellular senescence” in both O-linked and N-linked glycosylation-pathway^high^ chondrocytes (**Figure 2I**).

These results indicate that the cellular senescence signal is prominently connected to the O-linked glycosylation pathway rather than the N-linked glycosylation in OA chondrocytes and vice versa whenever taken together.

### 3.3 Expression analysis of DEGs linked to glycosylation pathway in OA chondrocytes

To further elucidate the contribution of the protein glycosylation pathway to the OA phenotype, we next identified DEGs related to the glycosylation pathway in OA chondrocytes using two scRNA-seq datasets (GSE104782 and GSE169454) (26, 27).

**Figure 3:**
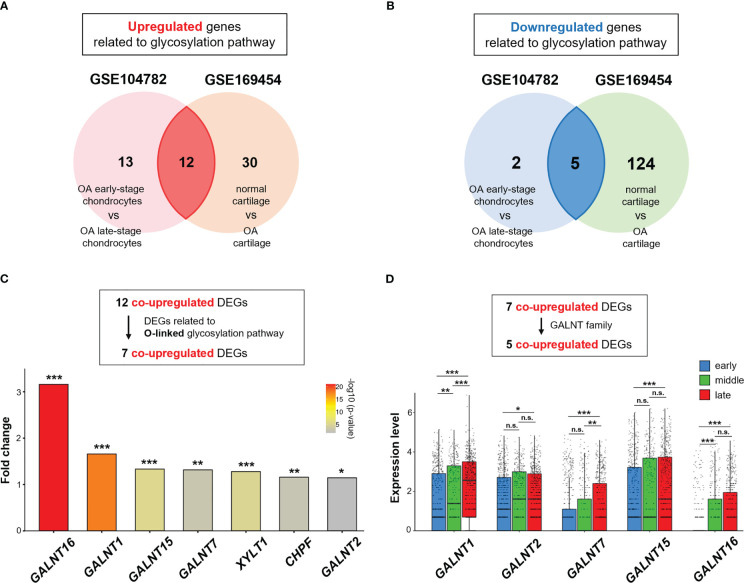
GALNT family gene expression is upregulated in OA chondrocytes. **(A, B)** Venn diagram showing overlapping differentially expressed genes (DEGs) in GSE104782 (OA chondrocytes at the early stage [grade 0; *n* = 262, and grade 1; *n* = 302] *vs*. OA chondrocytes at the late stage [grade 3; *n* = 284, and grade 4; *n* = 304], expressed in more than 30% of chondrocytes) and GSE169454 (normal cartilage; *n* = 8887 *vs.* OA cartilage; *n* = 57908). **(A)** Upregulated genes related to the glycosylation pathway in each dataset. **(B)** Downregulated genes related to the glycosylation pathway in each dataset. **(C)** Fold-change in upregulated DEGs related to the O-linked glycosylation pathway between OA chondrocytes at the late stage and early stages in the GSE104782 dataset (*P < 0.05, **P < 0.01, ***P < 0.001). **(D)** Expression of *GALNT1*, *GALNT2*, *GALNT7*, *GALNT15*, and *GALNT16* in each stage of OA chondrocytes in the GSE104782 dataset (*P < 0.05, **P < 0.01, ***P < 0.001, n.s., not significant).

Thirty-two and 171 DEGs were screened in GSE104782 (OA early-stage chondrocytes vs. OA late-stage chondrocytes) and GSE169454 (normal cartilage vs. OA cartilage), respectively. These include 25 and 42 significantly upregulated genes and 7 and 129 significantly downregulated genes in GSE104782 and GSE169454, respectively (**Figures 3A, B**).

Of the 25 and 42 DEGs in GSE104782 and GSE169454, respectively, the 12 overlapping genes were co-upregulated (**Figure 3A**), and 7 were related to the O-linked glycosylation (**Figure 3C**). Among the upregulated genes related to the O-linked glycosylation, polypeptide N-acetylgalactosaminyltransferase 16 (*GALNT16*), a member of the UDP-N-acetyl-α-D-galactosamine: polypeptide N-acetylgalactosaminyltransferase (GalNAc-T) family of enzymes essential for O-Glycan biosynthesis at the early stage (32–34), was the highest upregulated gene in OA chondrocytes at the late stage (grade 3 and 4) compared with those at the early stage (grade 0 and 1) (**Figure 3C**). Moreover, the expression levels of *GALNT1*, *GALNT2*, *GALNT7*, and *GALNT15* were significantly upregulated in OA chondrocytes at the late stage than at the early stage (**Figure 3C**). In addition, the expression of *GALNT1* in chondrocytes was positively associated with increased OA grades (early, middle, and late stages) (**Figure 3D**). Meanwhile, significantly increased expressions of *GALNT7* (at the late stage than at the middle stage) and *GALNT16* (the middle stage than at the early stage) were observed in chondrocytes (**Figure 3D**).

These results suggest that the O-linked glycosylation pathway and the corresponding genes (GALNT family) are activated in OA chondrocytes, and may play a role in OA pathophysiology.

### 3.4 Expression of the GALNT family is associated with pathological features of OA and chondrocyte senescence

Among the seven upregulated genes related to the O-linked glycosylation pathway (**Figure 3**), five significantly upregulated genes (*GALNT1*, *GALNT2*, *GALNT7*, *GALNT15*, and *GALNT16*) belonged to the GALNT family. Thus, we subsequently focused on the GALNT family.

**Figure 4:**
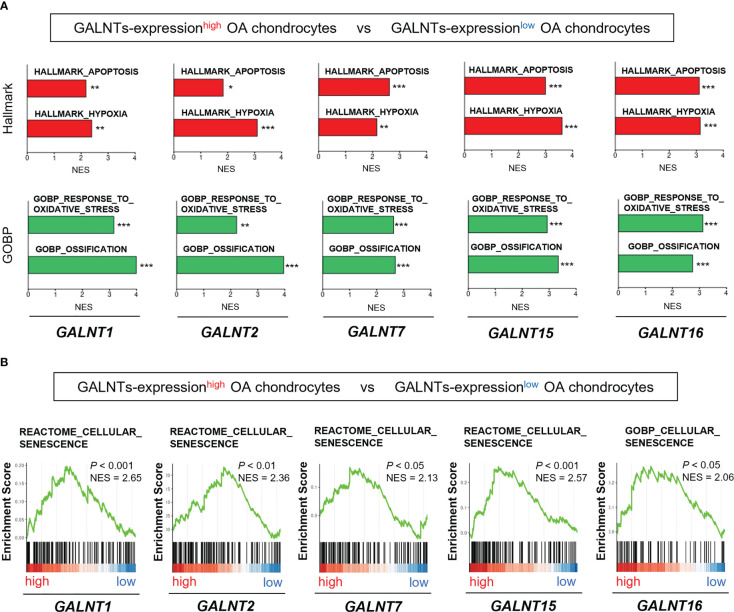
GALNT family gene expression is associated with the pathological features of OA and chondrocyte senescence. **(A, B)** GSEA of GALNTs-expression^high^ OA chondrocytes (the top 25% and bottom 25% of the expression level of the GALNTs-family in OA chondrocytes are defined as GALNTs-expression^high^ chondrocytes [*n* = 364] and GALNTs-expression^low^ chondrocytes [*n* = 364], respectively). **(A)** “apoptosis,” “hypoxia,” “oxidative stress,” and “ossification” gene sets (*P < 0.05, **P < 0.01, ***P < 0.001). **(B)** “cellular senescence” gene set.

We determined whether the expression of the GALNT family members was associated with the OA pathogenesis and chondrocyte senescence using the scRNA-seq dataset (GSE104782). We divided chondrocytes into GALNTs-expression^high^ and GALNTs-expression^low^ groups based on their expression levels in chondrocytes obtained from all stages (grades 0 to 4). GSEA revealed that gene sets linked to OA pathogenesis (“apoptosis,” “hypoxia,” “oxidative stress,” and “ossification”) were significantly enriched in *GALNT1-*, *GALNT2-*, *GALNT7-*, *GALNT15-* and *GALNT16*-expression^high^ OA chondrocytes (**Figure 4A**). In addition, the gene set involved in “cellular senescence” was significantly enriched in *GALNT1-*, *GALNT2-*, *GALNT7-*, *GALNT15-* and *GALNT16*-expression^high^ OA chondrocytes (**Figure 4B**).

These findings indicate that the GALNTs, which are key enzymes to initiate mucin-type O-glycosylation, are linked to the chondrocyte senescence and OA pathology.

## Discussion

In this study, we performed integrated bioinformatic analysis using an independent cohort and demonstrated a possible relationship between the cellular senescence signaling and glycosylation pathway in chondrocytes in context of OA pathogenesis. OA affects not only the cartilage and chondrocytes but also joint tissues and related cells, including subchondral bone, synovium, mesenchymal stem cells, bone cells, fibroblasts, and immune cells (3, 35–37). As various tissues and cells within the joint contribute to OA development, the implication of their interactions in the OA pathophysiology should be considered. Moreover, the cellular heterogenicity of chondrocytes may contribute to the pathophysiology of OA as different populations of chondrocytes are present in the OA cartilage (38–40). To the best of our knowledge, this is the first study to show the relationship between chondrocyte senescence and O-linked glycosylation and its role in the pathophysiology of OA using integrated bioinformatics analyses.

O-linked N-acetylglucosamine (O-GlcNAc) protein modification stimulates chondrogenic differentiation and is increasingly observed in the cartilage of OA patients, indicating the implication of O-linked glycosylation in the normal cartilage homeostasis and pathological cartilage degenerative disease (24, 25). The GALNT family, also known as ppGalNAc-Ts or GalNAc-Ts, comprises 20 isoenzymes responsible for initiating mucin-type O-glycosylation by transferring N-acetyl galactosamine to the hydroxyl group of a serine/threonine residue in Golgi apparatus (34). Although the GALNT family is highly homologous, individual GALNTs have different activities with different substrate specificities, and consequently, initiation of O-glycosylation is regulated by a repertoire of GALNTs (32, 33). The tumor growth and metastasis could be associated with the aberrant expression of GALNT family members, and distribution and subsequent marked alterations in GalNAc O-linked glycosylation in various cancer types (34, 41, 42). Moreover, the GALNT family is associated with various diseases, such as obesity, diabetes, and osteoporosis, in addition to CDGs (43–45). However, the role of the GALNT enzyme family in OA development and progression remains unknown. Here, we demonstrated that the expression levels of the GALNT enzyme family were significantly upregulated in OA chondrocytes, with *GALNT16* being the top upregulated gene, along with significant upregulation of *GALNT1*, *GALNT2*, *GALNT7*, and *GALNT15* among genes related to the O-linked glycosylation pathway. Notably, expression analysis of DEGs related to the glycosylation pathways revealed the involvement of alternative candidate genes in OA chondrocytes (**Figure 3**). Despite these additional genes and molecular pathways that need further exploration, we revealed the possible crucial connection between cellular senescence and O-linked glycosylation in OA chondrocytes.

Medications for OA, including pain relievers and non-steroidal anti-inflammatory drugs, are commonly prescribed to control its symptoms including chronic joint pain and stiffness (46, 47). Currently, DMOADs remain unavailable and thus, targeting cellular senescence and SASP with senotherapeutics, senolytic, and senomorphics drugs, could be beneficial in treating OA (48, 49). Our findings improve our understanding of the molecular mechanism underlying OA development and suggest that the relationship between cellular senescence signal and O-linked glycosylation status in chondrocytes can represent a novel and effective target for drug development to treat OA in humans.

## Data availability statement

The original contributions presented in the study are included in the article/supplementary material. Further inquiries can be directed to the corresponding author.

## Author contributions

MY, KS, and EH conceived the project. MY, KS, KT, YT, and KF performed the experiments and data analysis. MY and EH wrote the manuscript. All authors contributed to the article and approved the submitted version.
